# Mechanisms of sexually transmitted infection‐induced inflammation in women: implications for HIV risk

**DOI:** 10.1002/jia2.25346

**Published:** 2019-08-30

**Authors:** Ruth Mwatelah, Lyle R McKinnon, Cheryl Baxter, Quarraisha Abdool Karim, Salim S Abdool Karim

**Affiliations:** ^1^ Department of Medical Microbiology and Infectious Diseases University of Manitoba Winnipeg Canada; ^2^ Centre for the AIDS Programme of Research in South Africa (CAPRISA) University of KwaZulu‐Natal Durban South Africa; ^3^ Department of Epidemiology Columbia University New York NY USA

**Keywords:** immunology, inflammation, sexually transmitted infections, women, HIV, bacterial vaginosis, mucosal immune responses, adaptive immune responses

## Abstract

**Introduction:**

Globally, sexually transmitted infections (STI) affect >300 million people annually, and are a major cause of sexual and reproductive health complications in women. In this commentary, we describe how STIs interact with the immune and non‐immune cells, both within and below the cervicovaginal mucosal barrier, to cause inflammation, which in turn has been associated with increased HIV acquisition risk.

**Discussion:**

STIs have a major impact on the female genital mucosa, which is an important biological and physical barrier that forms the first line of defence against invading microorganisms such as HIV. Pattern recognition of STI pathogens, by receptors expressed either on the cell surface or inside the cell, typically triggers inflammation at the mucosal barrier. The types of mucosal responses vary by STI, and can be asymptomatic or culminate in the formation of discharge, ulcers and/or warts. While the aim of this response is to clear the invading microbes, in many cases these responses are either evaded or cause pathology that impairs barrier integrity and increases HIV access to target cells in the sub‐mucosa. In addition, innate responses to STIs can result in an increased number of immune cells, including those that are the primary targets of HIV, and may contribute to the association between STIs and increased susceptibility to HIV acquisition. Many of these cells are mediators of adaptive immunity, including tissue‐resident cells that may also display innate‐like functions. Bacterial vaginosis (BV) is another common cause of inflammation, and evidence for multiple interactions between BV, STIs and HIV suggest that susceptibility to these conditions should be considered in concert.

**Conclusions:**

STIs and other microbes can induce inflammation in the genital tract, perturbing the normal robust function of the mucosal barrier against HIV. While the impact of STIs on the mucosal immune system and HIV acquisition is often under‐appreciated, understanding their interactions of the infections with the immune responses play an important role in improving treatment and reducing the risk of HIV acquisition. The frequent sub‐clinical inflammation associated with STIs underscores the need for better STI diagnostics to reverse the immunological consequences of infection.

## 1 Introduction

There are over 50 types of viruses, bacteria and parasites that can be sexually transmitted, eight of which are most widely recognized as sexually transmitted infections (STIs). These include syphilis, gonorrhoea, chlamydia, trichomoniasis, hepatitis B, herpes simplex virus (HSV), human papillomavirus (HPV) and HIV 1. Most often, STIs are either asymptomatic or mildly symptomatic, and therefore remain undiagnosed and under‐recognized by patients and clinicians 2. Long‐term infection by STIs can cause severe reproductive health complications in women, including still birth, preterm delivery, increased risk of HIV acquisition, infertility and cancer, among others 3, 4, 5. In most high‐income countries, policies ensure the availability of diagnostic tests, rapid delivery of results and contact tracing for those infected; however, these are not typically available in low‐ and middle‐income countries where STIs are common and managed primarily on the basis of signs and symptoms. While syndromic diagnosis is reasonably sensitive and specific for ulcerative infections, other STIs are often missed due to poor sensitivity and may remain untreated for long periods of time 6.

Another challenge is drug resistance; infections such as *Neisseria gonorrhoeae* are increasingly resistant to standard therapies including macrolides, tetracyclines and cephalosporins 7. This increase in antibiotic resistance, combined with high prevalence, low rates of treatment, and their association with HIV transmission and reproductive compilations, all underscore the need to better understand the mucosal immune responses to STI‐causing organisms.

The purpose of this commentary was to describe how STIs interact with the vaginal mucosal barrier, and the commensal microbes that line its luminal surface, to cause inflammation. While this commentary focuses on STIs in women, some similar mechanisms have been suggested for male genital immunology 8, 9, 10. Many of the pathological effects of STIs correspond to biological mechanisms that may favour HIV acquisition in women.

## 2 Discussion

### 2.1 Types of mucosal immune responses to STIs

There are several ways to classify STIs, the most obvious being by the type of causative organism, that is, bacterial, viral or parasitic. A second important way is by clinical presentation; although STIs are frequently asymptomatic, they can also cause (a) ulcers in genital, anal, oral and perianal tissues (e.g. *Treponema pallidum*, HSV), (b) urethral and vaginal discharge (e.g. *Chlamydia trachomatis*,* N. gonorrhoeae* and *Mycoplasma genitalium*), or (c) genital warts (e.g. HPV) 11.

Yet another way to classify STIs is by the different mechanisms through which they cause infections and evade immunity. STIs result in a large inflammatory response that can lead to pathology throughout the genital tract, including pelvic inflammatory disease, ectopic pregnancy and infertility, and degradation of the epithelium. As part of this inflammatory response, an influx of immune cells including neutrophils has been associated with discharge and lesions in the genital tract, resulting in further damage to the epithelial barrier 12. We and others have shown that this epithelial damage may be due to increased protease expression, which functions to degrade epithelial integrity 13, 14.

Although the mechanisms differ, the ability of all STI‐causing pathogens to induce an inflammatory response, damage the epithelial barrier, and impair natural innate defences is believed to increase the risk of HIV acquisition, by providing the virus better access to HIV target cells in the sub‐mucosa and beyond. Inflammation may simultaneously increase the number of and location of these cells relative to the lumen or induce phenotypic changes that increase their cellular susceptibility to virus infection 15. The inflammatory responses induced by STIs is intended to (and in some cases may) play an important role in protecting the host, but in many other cases this response favours the pathogen. This could be due to evasion of the effector mechanisms that are aimed at pathogen clearance (see Table 1), but also by causing collateral damage to host tissues 16, 17, 18. For example, in *C. trachomatis* infection, neutrophils are among the first immune cells to be recruited to the site of infection. Delayed apoptosis is a strategy used by *C. trachomatis* to avoid a complete immune response whereby it reduces the neutrophil sensitivity towards the stimuli from apoptosis, hence contributing towards pathogen persistence 19.

**Table 1:** Immune evasion strategies employed by common STIs

Strategy	Definition	Examples	References
Internalization	Epithelial cell entry, avoiding extracellular mechanisms of immune surveillance such as antibody responses	*Chlamydia trachomatis Neisseria gonorrhoeae Mycoplasma genitalium*	26, 90, 91
Deregulation of cellular process	Inhibition of important cellular processes in order to dampen the immune response e.g. DNA methylation, maturation of DCs, activation of immunoinhibitory pathways	HPV, HSV2, *C. trachomatis Treponema* pallidum	92, 93, 94, 95
Resistance to antimicrobial peptides	Expression of genes which are highly resistant to antimicrobial peptides	*Haemophilus ducreyi*	96, 97, 98, 99
Interference with the processes of the complement system	Acquisition of CD59 from different host cells, which inhibits binding of C9 with C5b‐C8 that is critical for pore formation. In addition, this pathogen can stimulate iron induced cysteine protease activity.	*Trichomonas vaginalis*	100, 101
Structure alteration	Pathogen‐induced changes to their extracellular structure to avoid detection by the innate immune system.	*M. genitalium*	37
Inhibition of Th1 CD4 and CTL responses	Pathogens upregulate specific responses which results to the suppression other immune responses that would result to their clearance. For example, upregulation of Th17 response that results to the downregulation of Th1 response.	*M. genitalium, Chlamydia trachomatis T. vaginalis,* HSV2, HPV*, Treponema pallidum, N. gonorrhoeae*	95, 101, 102, 103, 104, 105, 106, 107, 108, 109, 110, 111, 112
Inhibition of other types of T cell responses (Th2, 17, 22, Treg)	The pathogen downregulates the immune response in specific cells like macrophages, dendritic cells and monocytes.	*T. vaginalis*, HPV*, N. gonorrhoeae*,* T. pallidum*	

### 2.2 STIs and genital inflammation

Genital inflammation, defined by elevated cytokines, has been a strong predictor of HIV acquisition risk and decreased TFV gel efficacy 20, 21. Elevated levels of inflammatory cytokines have been highly correlated to increased protease activity, which may decrease the integrity of the epithelial barrier 13, 14. South African women with laboratory‐confirmed STI infections had increased the levels of inflammatory cytokines in the genital tract, including IL‐1α, IL‐4, fractalkine, TNF‐β, macrophage‐derived chemokine, IL‐1β and interferon‐γ 20, 22. STIs have been associated with increased genital inflammation signatures specifically among those with *C. trachomatis* infections 23, 24, 25, 26. Many studies have established that mucosal cytokine production occurs after STI acquisition, forming a central feature of the ensuing immune response. Therefore, consideration of the broader immune pathways that drive these cytokine responses could provide important insight into how STIs change the mucosal milieu 27, 28.

### 2.3 Intracellular and extracellular recognition of STIs by pattern recognition receptors

**Figure 1:**
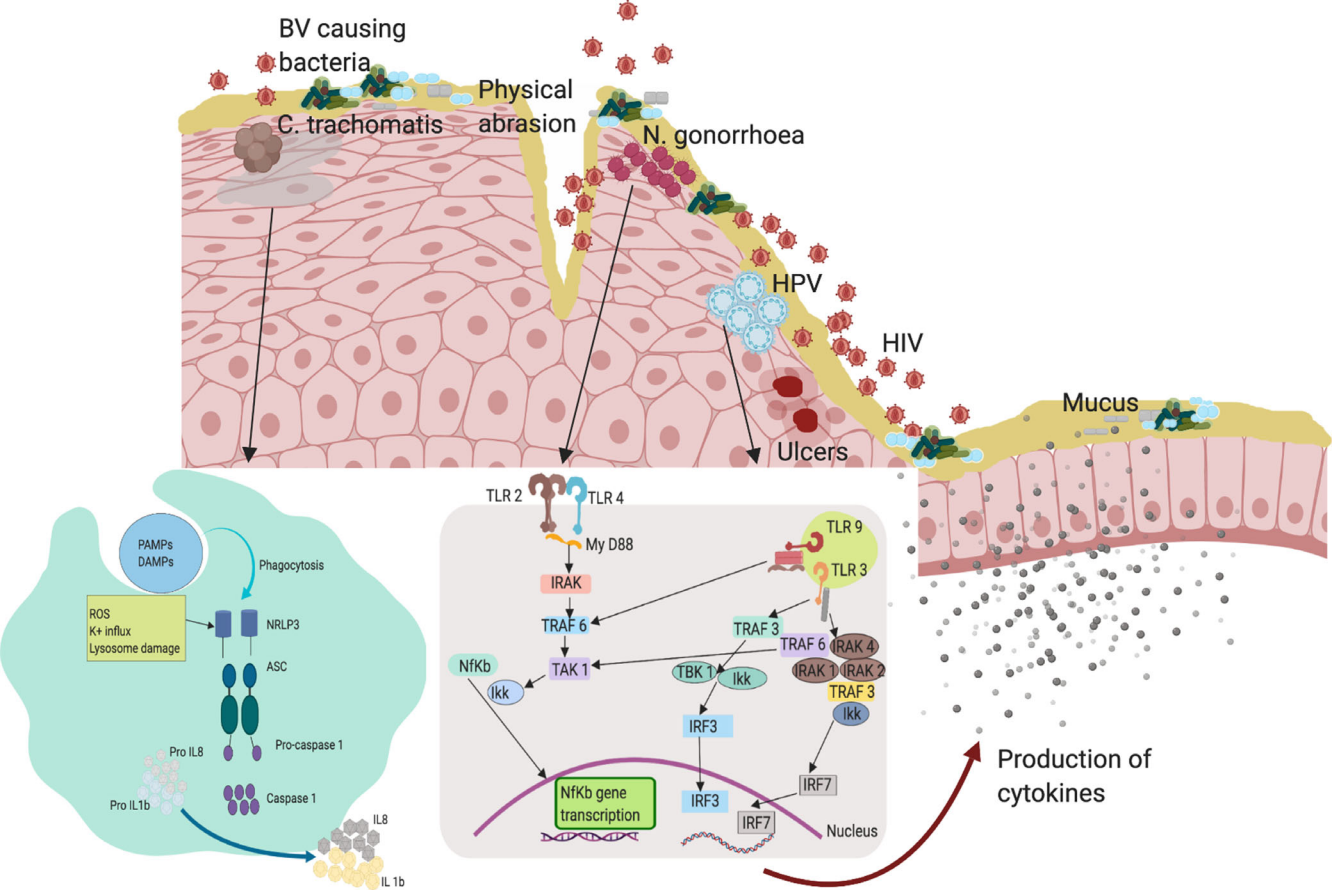
Mucosal innate immune responses to STIs in the female genital tract that could potentiate HIV transmission risk. Depicted are several of the modes through which STIs might increase the risk of HIV acquisition. Infection with STIs results to physical abrasion, ulcer formation and increase of pro‐inflammatory cytokines resulting in inflammation. Inflammation increases the availability of HIV target cells in the sub‐mucosa. During *N. gonorrhoeae* infection, TLR2 and 4 detect lipooligosaccharide and induce a NF‐KB driven immune response resulting to production of cytokines. Infection with *C. trachomatis* results in death of some cells which in turn produce elementary bodies. *C. trachomatis* infection is detected by inflammasomes resulting to production of IL‐1b and IL‐8 through the NLR3 pathway. TLR9 detects the CpG island in Genomic material of the HPV virus inducing an immune response through the MYD88 pathway. TLR3 detects the viral nucleic acid to induce an immune response through the IRF and IR7 pathways.

Mucosal epithelial cells are the first barrier against infection, forming an early line of defence against pathogen invasion. Epithelial cells are equipped with receptors that are crucial for pathogen detection, and these cells function to initiate and modulate the inflammatory cascade aimed at inducing pathogen clearance 29, 30. Inflammation leads to a series of reactions which induce adaptive immunity, including effector mechanisms that can clear infection. However, tight regulation of inflammation is required in order to avoid self‐damage 30, 31. In the case of STIs, a combination of immune evasion, potent induction of inflammation and poor natural immunity represents scenarios in which HIV entry may be increased (Figure 1).

Toll‐like receptors (TLRs) play an important role in detecting pathogens including STIs, and initiating appropriate innate and adaptive immune responses. TLRs bind to their cognate ligands, resulting in a signalling cascade that culminates in the expression of pro‐inflammatory cytokines. TLRs can be classified as both intracellular (TLR3,7,8,9,11,12 and 13) and extracellular (TLR1,2,4,5,6 and 10), on the basis of their expression and where ligand recognition typically occurs 32, 33. TLRs recognize pathogen‐associated molecular patterns (PAMPs), including bacterial DNA, viral nucleic acid and viral proteins, with the eventual goal of inducing specific T‐cell and antibody responses. For example, TLR9 detects the unmethylated CpG sequences in bacterial DNA molecules. Many TLRs signal via MyD88, an important intracellular protein adaptor molecule. MyD88 is responsible for induction of the IL‐1 family, a group of 11 mainly inflammatory cytokines that regulate innate immune cell function. IL‐1R‐associated kinase is recruited via MyD88 activation, further activating the NF‐kB pathway culminating in transcription of pro‐inflammatory cytokine genes 34, 35.

Several bacterial STIs induce innate inflammatory responses by interacting with extracellular TLRs. A recent study that utilized a 3D model of endocervical cells showed that *M. genitalium* was recognized by TLR2, 4 and 6, a pattern of TLR usage that initiates the NF‐KB pathway and is unique to this bacterium 36, 37. In microorganisms such as *Neisseria*, protein elements are detected both intracellularly and extracellularly, both of which can induce an NF‐KB driven inflammatory response. TLR 2 and 4 detect LPS, outer membrane vesicles, porins and other proteins, while additional pattern recognition molecules called NOD 1 and 2 detect additional STI biochemical structures such as gamma glutamyl diaminopimelic acid and muramyl dipeptide, which also results in induction of NF‐KB‐driven inflammation 38, 39.

Intracellular TLRs mainly detect viral infections. In contrast to many extracellular TLRs, which tend to recognize protein structures, intracellular expression of TLR3, TLR7, TLR8 and TLR9 mediates viral nucleic acid sensing. In a recent study that evaluated TLR gene expression by qPCR in endocervical cells of women, increased levels of TLR and IFN‐α2 were observed among those who had cleared HPV‐16 infection, suggesting that TLR responses may be associated with viral clearance. Moreover, HPV‐16 may interfere with these responses, thus enhancing their persistence 40. In this study, TLR9 expression was upregulated during high‐risk HPV infection and was higher in HPV‐positive compared to HPV‐negative individuals, confirming that TLR9 plays an important role in the detection of CpG islands in the DNA motifs during HPV infection *in vivo* 41.

STIs similarly induce immune responses through inflammasomes (multi‐protein intracellular structures located in the cytosol). The inflammasome is activated by the signalling of PAMPs, DAMPs (damage associated molecular proteins), changes in the ion concentrations of cytosol and by extracellular adenosine triphosphate (ATP). Once activated, this molecular complex leads to expression of pro‐inflammatory cytokines and can also initiate an inflammatory form of cell death called pyroptosis 42, 43, 44. Activation of the inflammasome often occurs through NOD‐like receptors (NLRs, especially NLR3), which interacts with apoptosis‐associated speck‐like protein containing a CARD (ASC). This protein is located in the nucleus of macrophages and monocytes and is responsible for activating caspase‐1, which in turn cleaves and activates IL‐1β and IL‐8 44. *C. trachomatis*, co‐cultured with epithelial cells, were found to activate inflammasomes resulting in IL‐1β and IL‐8 production and activation of pyroptosis. An inactivated form of *C. trachomatis* was tested in the same model and was still found to lead to priming of the inflammasome, but without the resulting inflammatory response, implying that pathogen replication may be critical for cytokine induction 45. This inflammatory pathway also applies to other STIs; for example, the LPS of *N. gonorrhoeae* has been shown to harbour hexa‐acylated lipid A, which can activate the NRLP3 inflammasome 46. *H. ducreyi* elicits IL‐1β responses that are dependent on activation of caspase‐1, ‐5 and NLRP3 in both M1 and 2 macrophages 47. In viral STIs such as HPV, cytosolic viral DNA is detected by AIM2 inflammasome and IFI16, an intracellular DNA sensor, resulting in the production of IL‐1β and IFN‐β respectively. Blocking of AIM2 resulted in increased production of IFN‐β thus it has the ability to block the production of IFN‐β an important mediator of antiviral response 48.

### 2.4 Co‐infection with STIs, bacterial vaginosis and HIV

In addition to the mucosal barrier, the composition of the vaginal microbiome can play an important role in providing immune defence at the genital mucosa. In particular, women with certain *Lactobacillus*‐dominant communities are able to produce lactic acid and maintain a low mucosal pH, which inhibits the growth of pathogenic bacteria including STIs. In the absence of *Lactobacillus* spp., with the exception of *Lactobacillus iners*, a more diverse microbiome population is typical, which is often associated with bacterial vaginosis (BV). BV, defined either by Nugent scoring or using molecular methods 49, has been associated with an increased risk of both STI and HIV acquisition 50, 51, 52, 53, 54, 55, 56, 57. Both STIs and BV are associated with increased levels of inflammatory cytokines like IFN‐α2, IL‐1α, IL‐1β, TNF‐α, IFN‐γ and IL‐8 51, 58. Epithelial cells of the genital mucosa produce glycogen, an energy source that allows *Lactobacillus* spp. to flourish 59, 60, which has been suggested provide protection against *Chlamydia* infection 61.

Synergism between BV and STIs is in part through the production of metabolites by the BV causing bacteria, which are utilized by STIs as growth factors. An example is seen between BV and *C. trachomatis* infections. Bacterial species that produce tryptophan have been associated with the increased risk of *C. trachomatis* infection among women whereas Indoleamine‐2,3‐dehydrogenase 1 (IDO1) producing species have been associated with decreased risk. IDOL1 inhibits the availability of tryptophan. This shows that BV may play an important role in both first time and recurrent *C. trachomatis* infections 62, 63. Ziklo *et al*. found that chlamydia infection was associated with reduced IFN‐γ response. IL‐17 was also reduced among infected individuals, and this cytokine is important in boosting host defence and maintaining mucosal barrier. Therefore, the increased levels of kynurenine, the byproduct of tryptophan breakdown, is associated with increased risk of HIV acquisition 63, 64, 65. Studies have suggested that ethnicity and not metabolic mechanisms may also underlie the association between chlamydia and HIV 28, 66, 67. Increased risk of *Trichomonas vaginalis* acquisition has been associated with BV in both HIV‐positive and ‐negative women 68.

Durable and effective treatment of BV has been a major challenge for the field. Oral or topical metronidazole is effective in the short term, yet recurrence occurs among more that 50% of women within three to twelve months 50, 69, 70. However, periodic presumptive treatment has proven to be an effective method in reducing STI incidence 71, 72. This strengthens the case for a causal relationship between BV and STIs, and also suggests that reducing BV may help to reduce STI incidence.

STI co‐infection in HIV‐positive women, particularly by *N. gonorrhoeae* or HSV‐2, increases inflammatory responses and mucosal HIV shedding 22, 73, 74, 75. In addition to mucosal inflammatory response, STIs such as *N. gonorrhoeae* have been found to increase plasma viral load and reduce CD4 T‐cell counts, indicating that both STI and HIV act synergistically resulting in detrimental effects to the host. While studies have suggested that STI treatment could reduce HIV shedding and transmission 73, this may be a moot point in the era of effective antiretroviral therapy, which, if taken correctly, reduces HIV transmission almost completely 76.

### 2.5 Role of adaptive immune response in STIs

Mechanisms of immunity to STIs are poorly understood, forming an obvious barrier to vaccine development. Epidemiological evidence for immunity to Chlamydia has been shown in the context to treatment 77. While the mechanism for the immunity is unclear, *C. trachomatis* infection has been associated with the formation of follicles 78; the presence of IFN‐γ+ CD4+ T cells in these follicles has been thought to provide an immune response in the case of a secondary infection 79. In some STI infections, re‐infection occurs long after the primary infection 80, 81, 82, as the adaptive immune response following primary infection plays a major role in immune surveillance and forms the first line of immune response in secondary infection.

Memory T‐cells were initially divided into central and effector memory T‐cells, which preferentially home to non‐lymphoid and secondary lymphoid organs respectively. Since that time, it is clear that there is an additional population of tissue‐resident memory lymphocytes that either do not re‐circulate, or re‐circulate very slowly, and provide rapid responses to re‐infection 83, 84. The role of these cells in the STI response is only beginning to be explored, with some data emerging for HSV‐2. In HSV‐2, a persistent infection occurs at the dermal epidermal joint (DEJ) of the mucosal lining with CD8+ T cells being the most predominant immune cells at this site. An assessment of CD8+ T‐cells at the DEJ in biopsies of HSV‐2 infected individuals revealed a high proportion of CD8 TCRαβ T‐cells. A comparison of the prevalence of CD8β or CD8α subsets at the DEJ showed that there was a higher population of CD8α mRNA, which were specifically CD8αα homodimers, an indication that they are responsible for containing HSV‐2 infection. The CD8α T‐cells formed clusters around epithelial cells that were HSV‐2 specific 85.

Additional cells including mucosal associated invariant T (MAIT) cells, invariant natural killer T (iNKT) cells, γδ T‐cells, innate lymphoid cells and IELs form part of the connection between the innate and adaptive response, and play a major role in guarding the integrity of the tissue and generation of local immune responses. Some studies support the presence of these cells in the vagina 86, 87, 88, 89, however, their responses to STIs have not been extensively explored.

## 3 Conclusions

In summary, STIs induce inflammatory responses through interactions with the epithelial barrier and immune cells at the site of infection. There are several molecular pathways involved in the inflammatory response to a diverse range of STIs, all of which likely function to cause pathology by weakening the mucosal barrier. At the same time, STIs use a variety of immune evasion strategies to dampen the immune response and enhance their persistence. STIs and BV likely both increase the risk of HIV acquisition by damaging the mucosal barrier and increasing pro‐inflammatory cytokines, increasing the availability of HIV target cells. The impact of STIs on mucosal immune responses and HIV acquisition is often under‐appreciated, but improved control of these infections through better diagnosis, treatment and prevention could make an important contribution to reducing HIV risk and improving reproductive health outcomes.

## Competing interests

The authors declare no conflicts of interest.

## Authors’ contributions

RM wrote the first draft of the paper. LRM, CB, SSAK and QAK provided critical review of the paper.
